# Supplementary material for: ChatGPT as a Tool for Biostatisticians: A Tutorial on Applications, Opportunities, and Limitations
Source: Stat Med. 2025 Oct 23;44(23-24):e70263. doi: 10.1002/sim.70263 (PMC12548020; doi:10.1002/sim.70263)
Supplement: Supplementary file 1 — Data S1. Supporting Information. [file SIM-44-0-s001.zip › Supplementary Materials Additional.zip!/3.7_Translation_Between_Multiple_Statistical_Programming_Languages/3.7.1_R_to_python/3.7.1_Translation_Chat8.pdf]

Run 8

Clear memory

I don't actually have memory on in this chat, so I can't remember anything you've told me in previous conversations or save anything for the future.

If you'd like me to remember things going forward (like your preferences or goals), you can turn memory on in **Settings > Personalization > Memory**.

Let me know if you want help with that!

Take the role of an expert biostatistician for this chat

Got it — I'm now your expert biostatistician. Whether you're working on clinical trial design, survival analysis, mixed models, or just trying to clean messy data in R or SAS, I've got you covered. What are we diving into today?

Translate the following code into python:

```
library(MASS)
set.seed(1)
temp <- tempfile()
download.file(
  "https://archive.ics.uci.edu/static/public/17/breast+cancer+wisconsin+diagnostic.zip",
  temp
)
data <- read.table(unz(temp, "wdbc.data"), sep = ",")
data$V2 <- data$V2 == "M"
unlink(temp)

train_index <- sample(seq_len(nrow(data)), round(nrow(data) * 0.8))

train <- data[train_index, -1]
test <- data[-train_index, -1]

base_model <- glm(V2 ~ 1, data = train, family = binomial)


step_model <- stepAIC(
  base_model,
```

```
  direction = "both",
  scope = list(
    lower = V2 ~ 1,
    upper = V2 ~ V3 + V4 + V5 + V6 + V7 + V8 + V9 + V10 + V11 +
      V12 + V13 + V14 + V15 + V16 + V17 + V18 + V19 + V20 + V21 +
      V22 + V23 + V24 + V25 + V26 + V27 + V28 + V29 + V30 + V31
  ),
  # scope = formula(V2 ~ .),
)

summary(step_model)
test_pred <- predict(step_model, test, type = "response")

sum((test_pred > 0.5) == test$V2) / nrow (test)
names(coef(step_model))
```

Here's the Python translation of your R code using **`pandas`**, **`numpy`**, **`sklearn`**, and **`statsmodels`** to closely replicate the functionality of **`stepAIC`** from the **`MASS`** package:

```python
import pandas as pd
import numpy as np
import statsmodels.api as sm
from sklearn.model_selection import train_test_split
from urllib.request import urlopen
import zipfile
import io

# Download and load data
url = "https://archive.ics.uci.edu/static/public/17/breast+cancer+wisconsin+diagnostic.zip"
with urlopen(url) as zipresp:
    with zipfile.ZipFile(io.BytesIO(zipresp.read())) as zfile:
        with zfile.open("wdbc.data") as f:
            data = pd.read_csv(f, header=None)

# Recode diagnosis column to binary
data[1] = data[1] == 'M'

# Train-test split
train_data, test_data = train_test_split(data, test_size=0.2, random_state=1)

# Define predictors and outcome
X_train = train_data.iloc[:, 2:]
y_train = train_data[1].astype(int)
X_test = test_data.iloc[:, 2:]
y_test = test_data[1].astype(int)

# Start with intercept-only model
X_base = sm.add_constant(pd.DataFrame(index=X_train.index))
base_model = sm.Logit(y_train, X_base).fit(disp=0)

# Stepwise selection
def stepwise_selection(X, y,
                       initial_features=[],
                       threshold_in=0.01,
                       threshold_out=0.05,
                       verbose=True):
    included = list(initial_features)
    while True:
        changed = False
        # Forward step
        excluded = list(set(X.columns) - set(included))
        new_pval = pd.Series(index=excluded, dtype=float)
```

```
        for new_column in excluded:
            model = sm.Logit(y, sm.add_constant(pd.DataFrame(X[included + [new_column]]))).fit(disp=
            new_pval[new_column] = model.pvalues[new_column]
        best_pval = new_pval.min()
        if best_pval < threshold_in:
            best_feature = new_pval.idxmin()
            included.append(best_feature)
            changed = True
            if verbose:
                print(f"Add {best_feature} (p={best_pval:.6})")
        # Backward step
        model = sm.Logit(y, sm.add_constant(pd.DataFrame(X[included]))).fit(disp=0)
        pvalues = model.pvalues.iloc[1:]  # exclude intercept
        worst_pval = pvalues.max()
        if worst_pval > threshold_out:
            worst_feature = pvalues.idxmax()
            included.remove(worst_feature)
            changed = True
            if verbose:
                print(f"Drop {worst_feature} (p={worst_pval:.6})")
        if not changed:
            break
    return included

selected_features = stepwise_selection(X_train, y_train)

# Final model
X_train_selected = sm.add_constant(X_train[selected_features])
final_model = sm.Logit(y_train, X_train_selected).fit()
print(final_model.summary())

# Predictions
X_test_selected = sm.add_constant(X_test[selected_features])
test_pred_probs = final_model.predict(X_test_selected)
test_pred = (test_pred_probs > 0.5).astype(int)

# Accuracy
accuracy = (test_pred == y_test).mean()
print("Test accuracy:", accuracy)

# Coefficient names
print("Selected features:", final_model.params.index.tolist())
```

Key Notes:

- R's `stepAIC` doesn't have a direct equivalent in Python, but the custom `stepwise_selection` function above is a commonly used approach.
- The intercept is manually handled using `sm.add_constant`.
- `Logit` from `statsmodels` matches R's `glm(..., family = binomial)`.
- The threshold values in stepwise selection can be tuned to match `AIC`-based behavior more closely.

Let me know if you'd like to visualize coefficients, ROC curve, or compare AIC/BIC metrics!

How can the thresholds be tuned to match AIC-based behavior?

Great question — tuning `threshold_in` and `threshold_out` in stepwise selection is essentially an approximation when you're trying to mimic AIC-based behavior like R's `stepAIC`. Here's how the two methods relate and how you can align them more closely:

🧠 Understanding the Difference

- **`stepAIC` in R**: Minimizes the Akaike Information Criterion (AIC), which balances model fit (likelihood) and complexity (number of parameters). Lower AIC is better.
- **Threshold-based stepwise selection in Python**: Uses *p-values* for adding/removing predictors based on statistical significance — not directly minimizing AIC.

So while p-values and AIC can often point to similar models, they aren't the same.

🔧 Aligning with AIC-Based Behavior

To mimic `stepAIC` more closely:

✅ Option 1: Use `AIC` directly (recommended)

Instead of relying on p-values, you can rewrite the `stepwise_selection` function to directly **track AIC**:

```python
def stepwise_selection_aic(X, y, verbose=True):
    included = []
    best_aic = np.inf
    while True:
        changed = False
        # Try adding
        excluded = list(set(X.columns) - set(included))
        add_aic = pd.Series(index=excluded, dtype=float)
        for new_col in excluded:
            try:
                model = sm.Logit(y, sm.add_constant(X[included + [new_col]])).fit(disp=0)
                add_aic[new_col] = model.aic
            except:
                continue
        if not add_aic.empty and add_aic.min() < best_aic:
            best_feature = add_aic.idxmin()
            best_aic = add_aic.min()
            included.append(best_feature)
            changed = True
            if verbose:
                print(f"Add {best_feature}, AIC: {best_aic:.2f}")

        # Try dropping
        if len(included) > 1:
            drop_aic = pd.Series(index=included, dtype=float)
            for col in included:
                trial_features = [f for f in included if f != col]
                model = sm.Logit(y, sm.add_constant(X[trial_features])).fit(disp=0)
                drop_aic[col] = model.aic
            if drop_aic.min() < best_aic:
                worst_feature = drop_aic.idxmin()
                best_aic = drop_aic.min()
                included.remove(worst_feature)
                changed = True
                if verbose:
                    print(f"Drop {worst_feature}, AIC: {best_aic:.2f}")
        if not changed:
            break
    return included
```

⚖️ Option 2: Tune thresholds (less precise)

If you stick with the p-value method:

- **Lower `threshold_in` (e.g., 0.01)** = more conservative, adds only strong predictors
- **Higher `threshold_out` (e.g., 0.10)** = more forgiving, allows weaker predictors to stay

But remember: p-values depend on the sample size and do **not** penalize complexity the way AIC does. So if the goal is true AIC minimization, go with Option 1.

Would you like me to run a quick comparison between the two methods on the same dataset?

Combine the stepwise_selection_aic with the rest of the code to provide a runnable script

Absolutely — here's a full, clean, **runnable Python script** that loads the Breast Cancer Wisconsin dataset, performs **stepwise logistic regression using AIC-based selection**, and evaluates accuracy on a test set:

```python
import pandas as pd
import numpy as np
import statsmodels.api as sm
from sklearn.model_selection import train_test_split
from urllib.request import urlopen
import zipfile
import io

# Download and extract the data
url = "https://archive.ics.uci.edu/static/public/17/breast+cancer+wisconsin+diagnostic.zip"
with urlopen(url) as zipresp:
    with zipfile.ZipFile(io.BytesIO(zipresp.read())) as zfile:
        with zfile.open("wdbc.data") as f:
            data = pd.read_csv(f, header=None)

# Recode diagnosis column (column 1): M = 1, B = 0
data[1] = data[1] == 'M'

# Train-test split
train_data, test_data = train_test_split(data, test_size=0.2, random_state=1)

# Define predictors and outcome
X_train = train_data.iloc[:, 2:]
y_train = train_data[1].astype(int)
X_test = test_data.iloc[:, 2:]
y_test = test_data[1].astype(int)

# Stepwise AIC-based selection
def stepwise_selection_aic(X, y, verbose=True):
    included = []
    best_aic = np.inf
    while True:
        changed = False
        # Try adding features
        excluded = list(set(X.columns) - set(included))
        add_aic = pd.Series(index=excluded, dtype=float)
        for new_col in excluded:
            try:
                model = sm.Logit(y, sm.add_constant(X[included + [new_col]])).fit(disp=0)
                add_aic[new_col] = model.aic
            except:
                continue
        if not add_aic.empty and add_aic.min() < best_aic:
            best_feature = add_aic.idxmin()
```

```
            best_aic = add_aic.min()
            included.append(best_feature)
            changed = True
            if verbose:
                print(f"Add {best_feature}, AIC: {best_aic:.2f}")

        # Try removing features
        if len(included) > 1:
            drop_aic = pd.Series(index=included, dtype=float)
            for col in included:
                trial_features = [f for f in included if f != col]
                try:
                    model = sm.Logit(y, sm.add_constant(X[trial_features])).fit(disp=0)
                    drop_aic[col] = model.aic
                except:
                    continue
            if not drop_aic.empty and drop_aic.min() < best_aic:
                worst_feature = drop_aic.idxmin()
                best_aic = drop_aic.min()
                included.remove(worst_feature)
                changed = True
                if verbose:
                    print(f"Drop {worst_feature}, AIC: {best_aic:.2f}")
        if not changed:
            break
    return included

# Run stepwise AIC selection
selected_features = stepwise_selection_aic(X_train, y_train)

# Fit final model
X_train_selected = sm.add_constant(X_train[selected_features])
final_model = sm.Logit(y_train, X_train_selected).fit()
print(final_model.summary())

# Predict on test data
X_test_selected = sm.add_constant(X_test[selected_features])
test_pred_probs = final_model.predict(X_test_selected)
test_pred = (test_pred_probs > 0.5).astype(int)

# Accuracy
accuracy = (test_pred == y_test).mean()
print("\nTest accuracy:", round(accuracy, 4))

# Final selected features
print("Selected features:", final_model.params.index.tolist())
```

✅ Output Summary:

- Step-by-step logging of features added/removed based on AIC.
- Full model summary with coefficients and p-values.
- Test set classification accuracy.
- Final feature set used in the model.

Let me know if you'd like to add ROC curves, confusion matrix, or cross-validation!
